# Supplementary material for: Predicting the Effect of Single Mutations on Protein Stability and Binding with Respect to Types of Mutations
Source: Int J Mol Sci. 2023 Jul 28;24(15):12073. doi: 10.3390/ijms241512073 (PMC10418460; doi:10.3390/ijms241512073)
Supplement: Supplementary file 1 [file ijms-24-12073-s001.zip › ijms-2527097-supplementary.pdf]

Predicting the effect of single mutations on protein stability and binding with respect to types of mutations

Preeti Pandey[1], Shailesh Kumar Panday[1], Prawin Rimal[1], Nicolas Ancona[2], Emil Alexov[1,*]

[1] Department of Physics and Astronomy, Clemson University, Clemson, SC 29634
[2] Department of Biological Sciences, Clemson University, Clemson, SC 29634
* Correspondance: ealexov@clemson.edu

Supplementary Figures

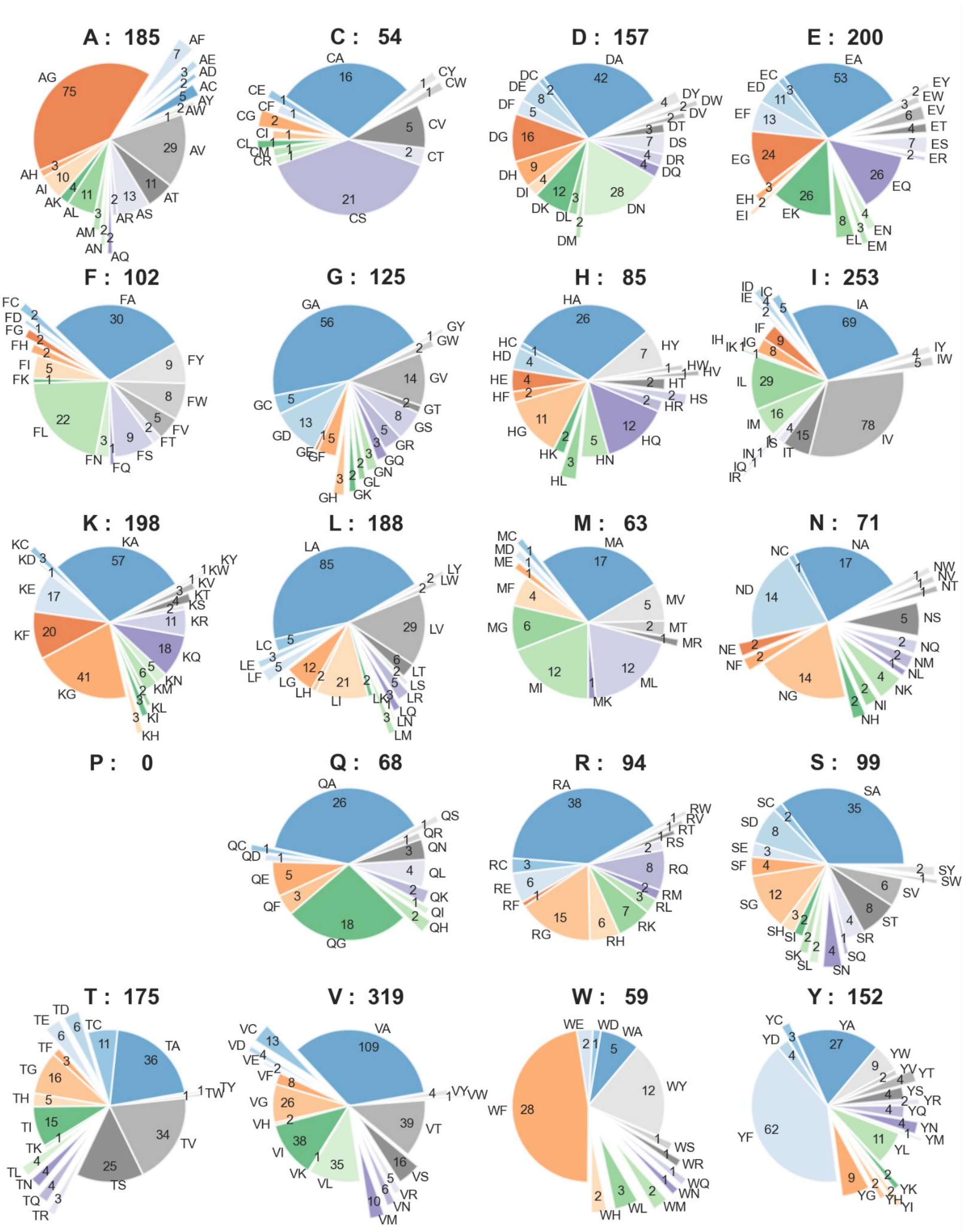

Figure S1a. Pie-chart summarizing count of different amino acid mutations in the S2648 dataset. In each subplot, the title shows the wild-type amino acids followed by their count in the whole dataset, and the two-letter label of every pie denotes the one-letter codes of the wild-type and mutant amino acid pair, respectively.

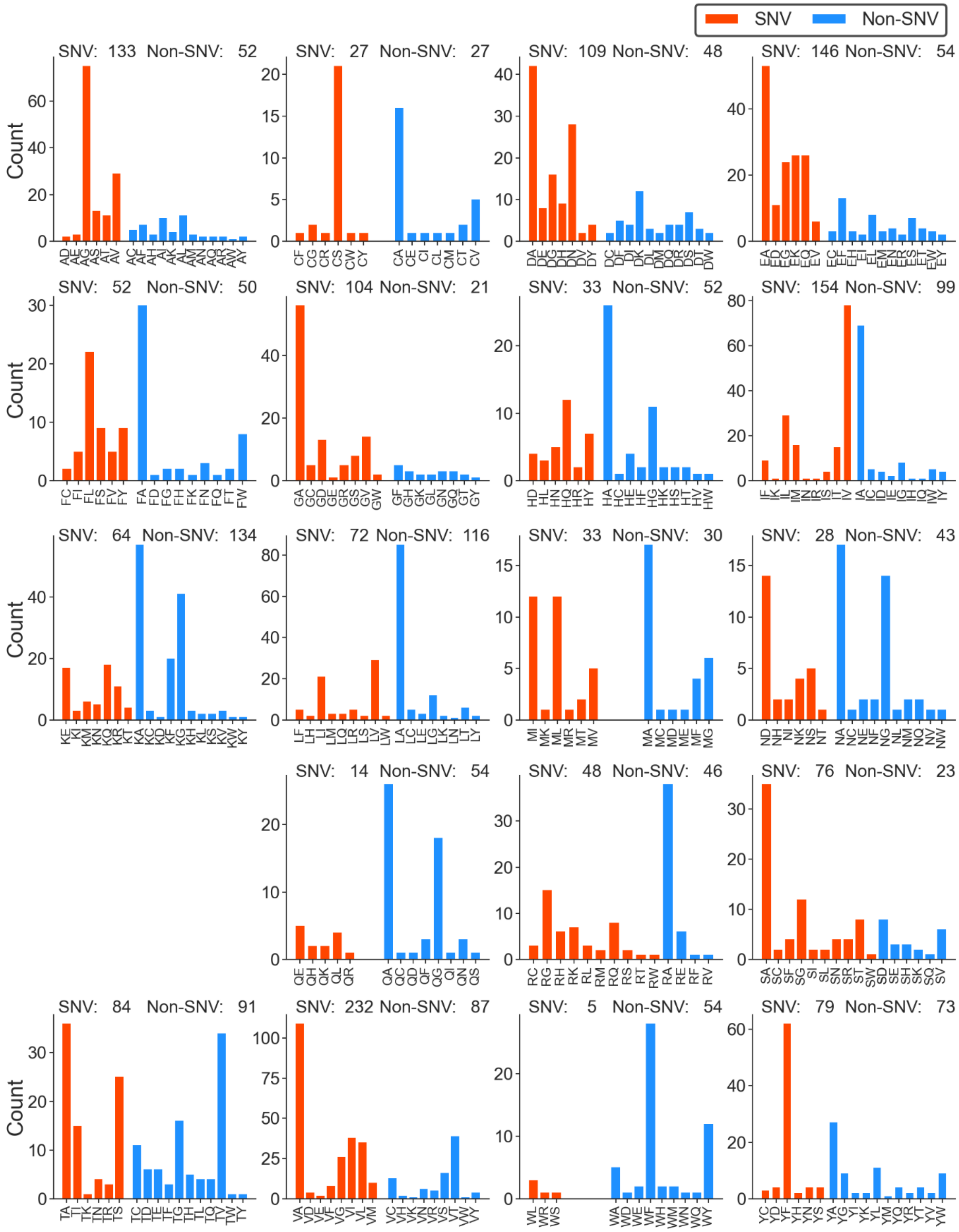

Figure S1b. Bar plots summarizing count of SNV and non-SNV mutations in the S2648 dataset. The two-letter label of every bar denotes the one-letter codes of the wild-type and mutant amino acid pairs, respectively.

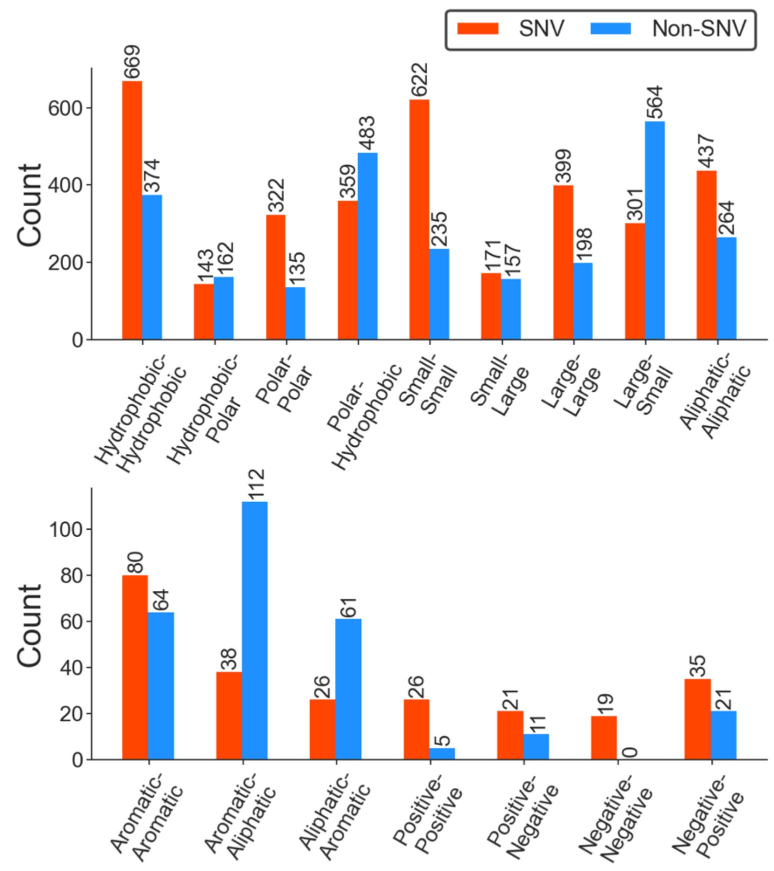

Figure S1c. SNV *vs* non-SNV mutations is assigned according to the property of amino acid mutation in the S2648 dataset. The two-word label of bars in the plot represents the amino acid properties of wild-type and mutant residues pairs, respectively. The amino acids according to their properties are categorized as follows: Ala, Cys, Gly, Ile, Leu, Met, Phe, Pro, Trp, and Val are categorized as hydrophobic. Asp, Glu, Lys, Arg, His, Asn, Gln, Ser, Thr, and Tyr are categorized as polar. His, Phe, Trp, and Tyr as aromatic. Ala, ILe, Lys, Leu, Met, Pro, and Val as aliphatic. His, Lys, Arg as positive. Asp, and Glu as negative. Ala, Cys, Gly, Ser, Asn, Asp, Pro, Thr, and Val as small. And Arg, Gln, Glu, His, ILe, Leu, Lys, Met, Phe, Trp, and Tyr as large amino acids.

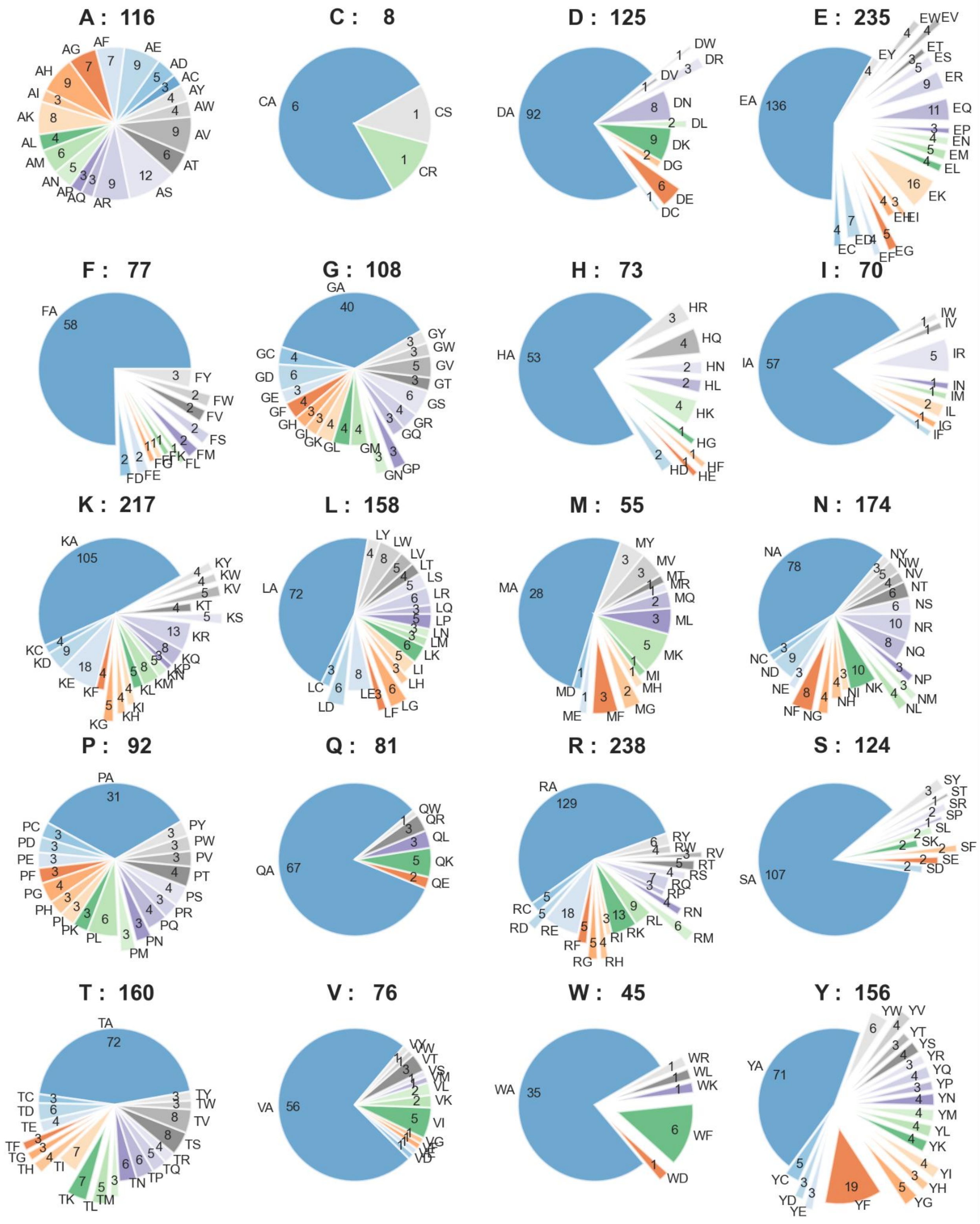

Figure S1d. Pie-chart summarizing count of different amino acid mutations in SKEMPI-SEQ-2388. In each subplot, the title shows the wild-type amino acids followed by their count in the whole dataset, and the two-letter label of every pie denotes the one-letter codes of the wild-type and mutant amino acid pair respectively.

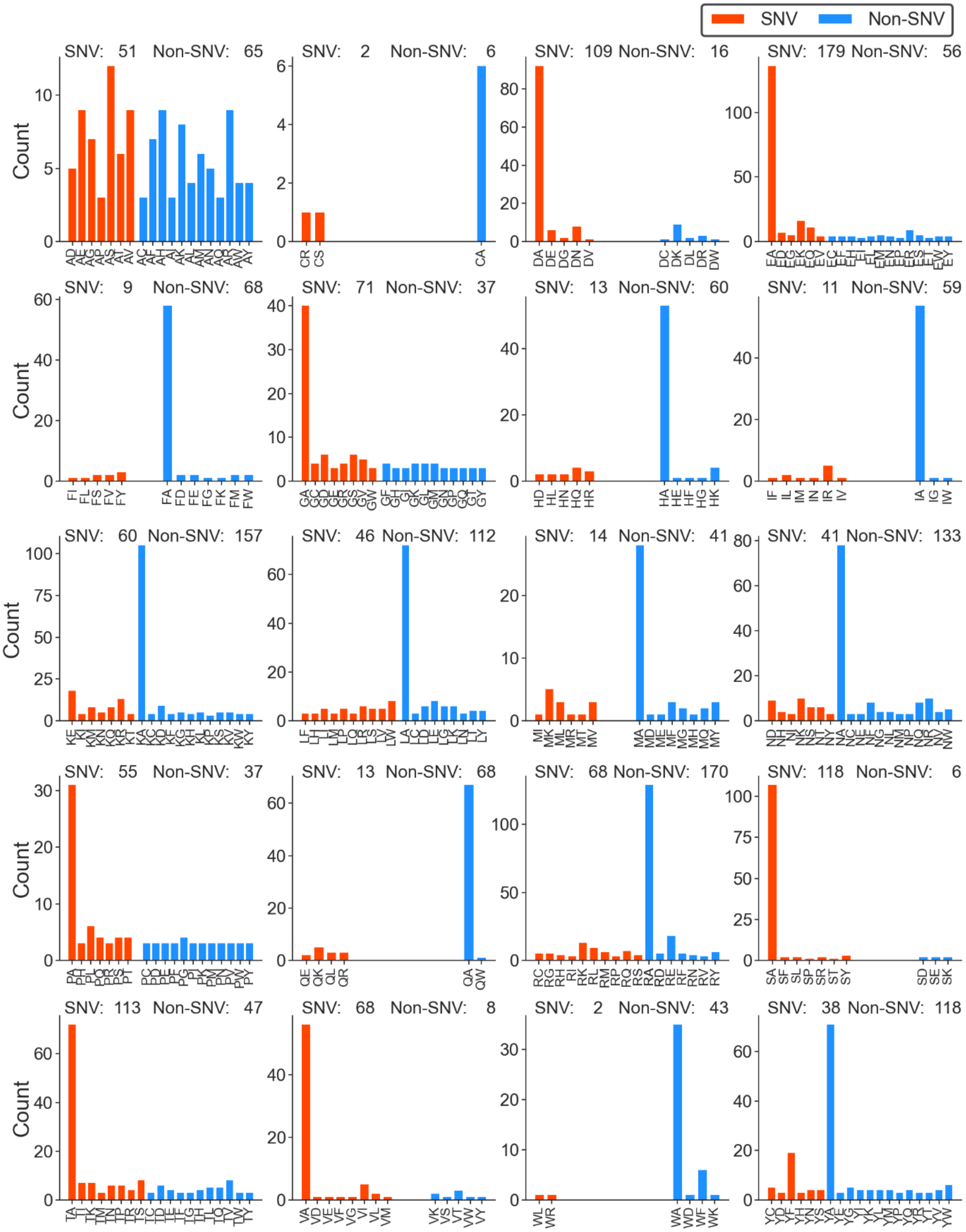

Figure S1e. Bar plot summarizing count of SNV and non-SNV mutations in SKEMPI-SEQ-2388 dataset. The two-letter label of every bar denotes the one-letter codes of the wild-type and mutant amino acid pairs, respectively.

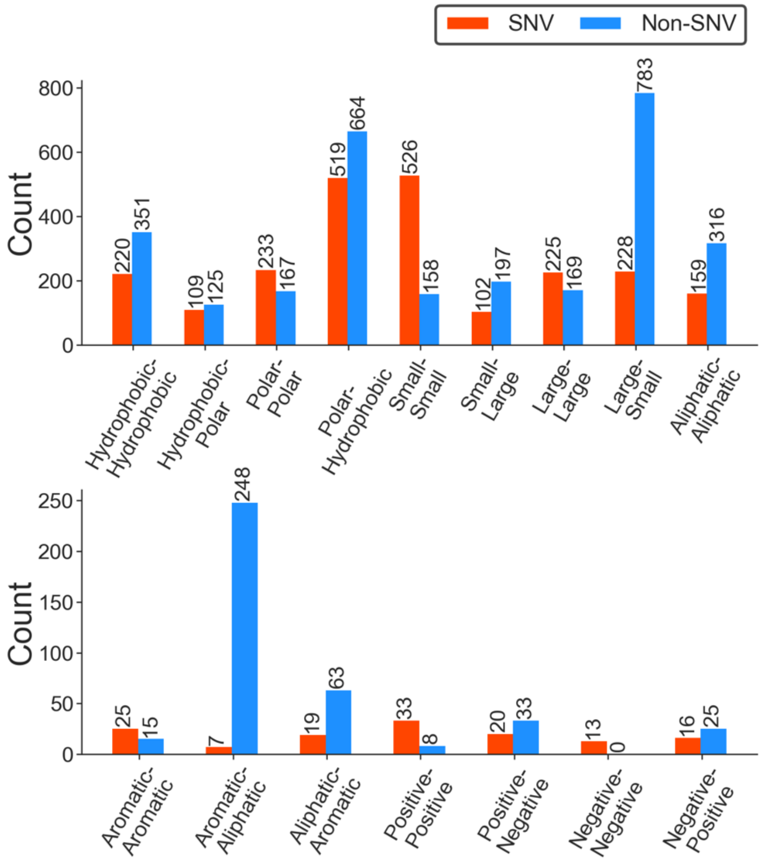

Figure S1f. SNV vs non-SNV mutations is assigned according to the type of amino acid mutation in the SKEMPI-SEQ-2388 dataset. The two-word label of bars in the plot represents the amino acid properties of wild-type and mutant residues pairs, respectively. The amino acids according to their properties are categorized as follows: Ala, Cys, Gly, Ile, Leu, Met, Phe, Pro, Trp, and Val are categorized as hydrophobic. Asp, Glu, Lys, Arg, His, Asn, Gln, Ser, Thr, and Tyr are categorized as polar. His, Phe, Trp, and Tyr as aromatic. Ala, ILe, Lys, Leu, Met, Pro, and Val as aliphatic. His, Lys, Arg as positive. Asp, and Glu as negative. Ala, Cys, Gly, Ser, Asn, Asp, Pro, Thr, and Val as small. And Arg, Gln, Glu, His, ILe, Leu, Lys, Met, Phe, Trp, and Tyr as large amino acids.

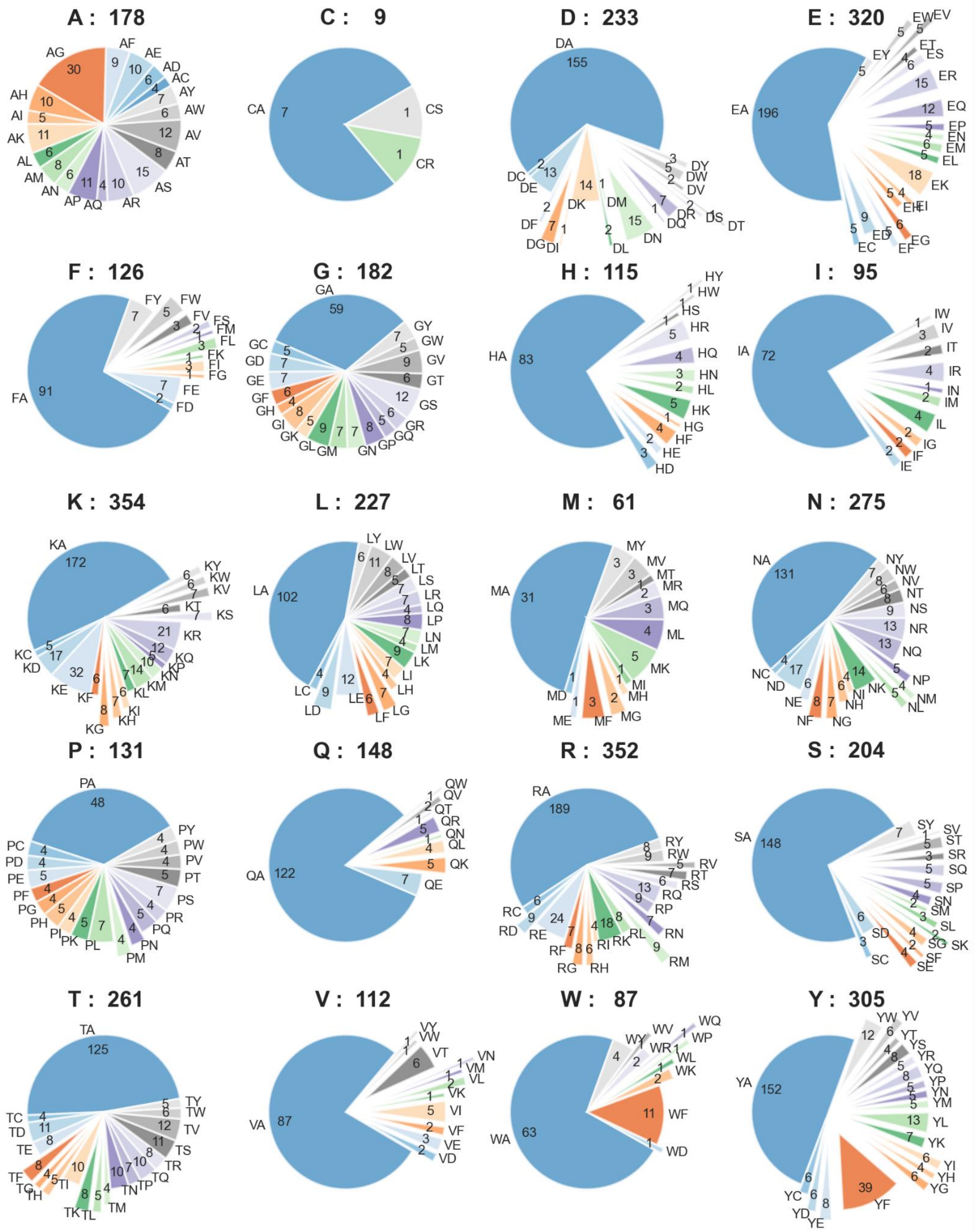

Figure S1g. Pie-chart summarizing count of different amino acid mutations in SKEMPI-3D-3775 dataset. In each subplot, the title shows the wild-type amino acids followed by their count in the whole dataset, and the two-letter label of every pie denotes the one-letter codes of the wild-type and mutant amino acid pair respectively.

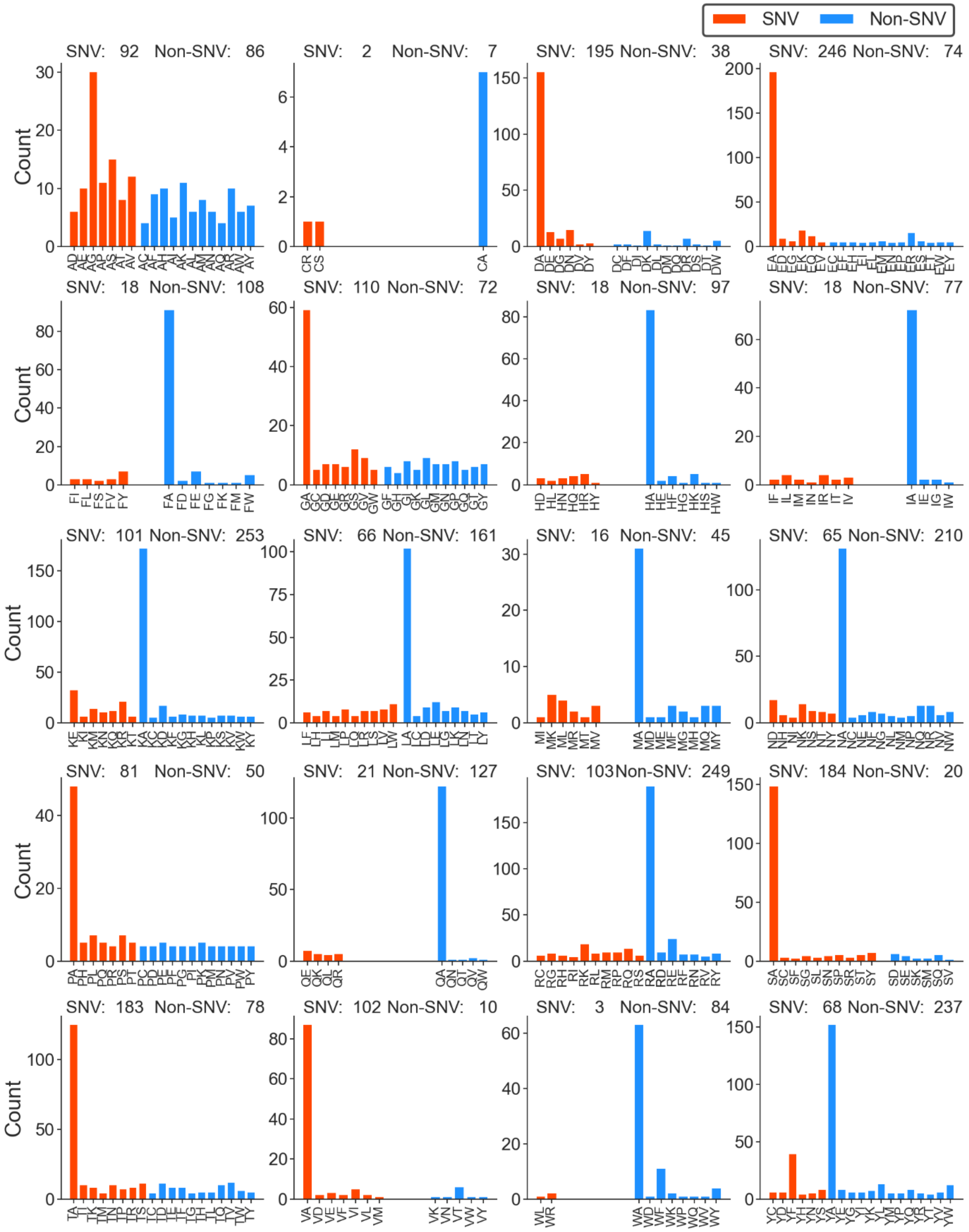

Figure S1h. Barplot summarizing count of SNV and non-SNV mutations in S2648 SKEMPI-3D-3775 dataset. The two-letter label of every bar denotes the one-letter codes of the wild-type and mutant amino acid pairs, respectively.

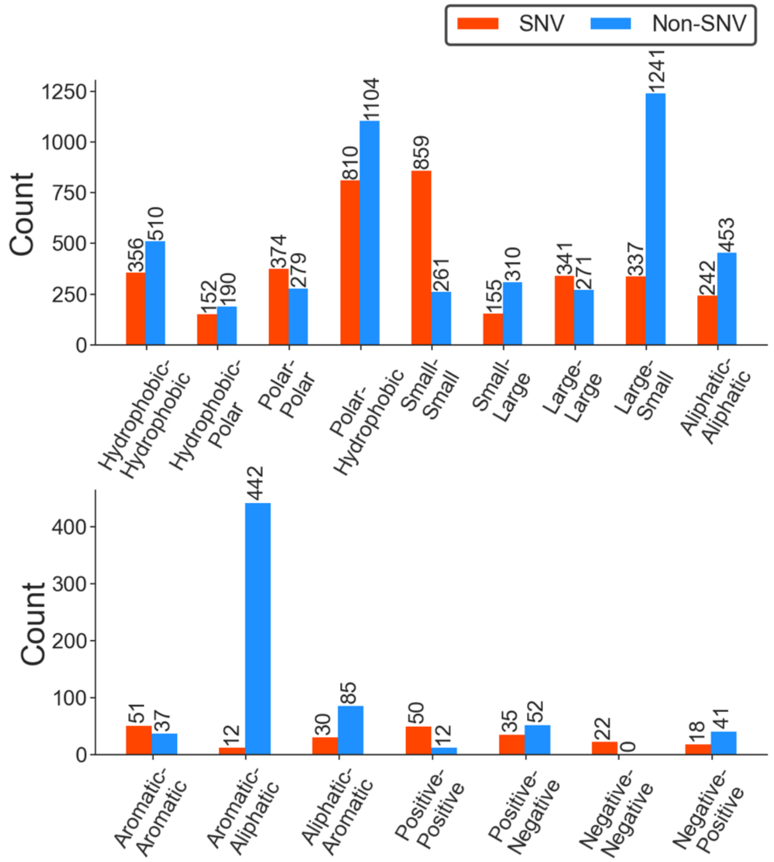

Figure S1i. SNV *vs* non-SNV mutations is assigned according to the type of amino acid mutation in the SKEMPI-3D-3775 dataset. The two-word label of bars in the plot represents the amino acid properties of wild-type and mutant residues pairs, respectively. The amino acids according to their properties are categorized as follows: Ala, Cys, Gly, Ile, Leu, Met, Phe, Pro, Trp, and Val are categorized as hydrophobic. Asp, Glu, Lys, Arg, His, Asn, Gln, Ser, Thr, and Tyr are categorized as polar. His, Phe, Trp, and Tyr as aromatic. Ala, ILe, Lys, Leu, Met, Pro, and Val as aliphatic. His, Lys, Arg as positive. Asp, and Glu as negative. Ala, Cys, Gly, Ser, Asn, Asp, Pro, Thr, and Val as small. And Arg, Gln, Glu, His, ILe, Leu, Lys, Met, Phe, Trp, and Tyr as large amino acids.

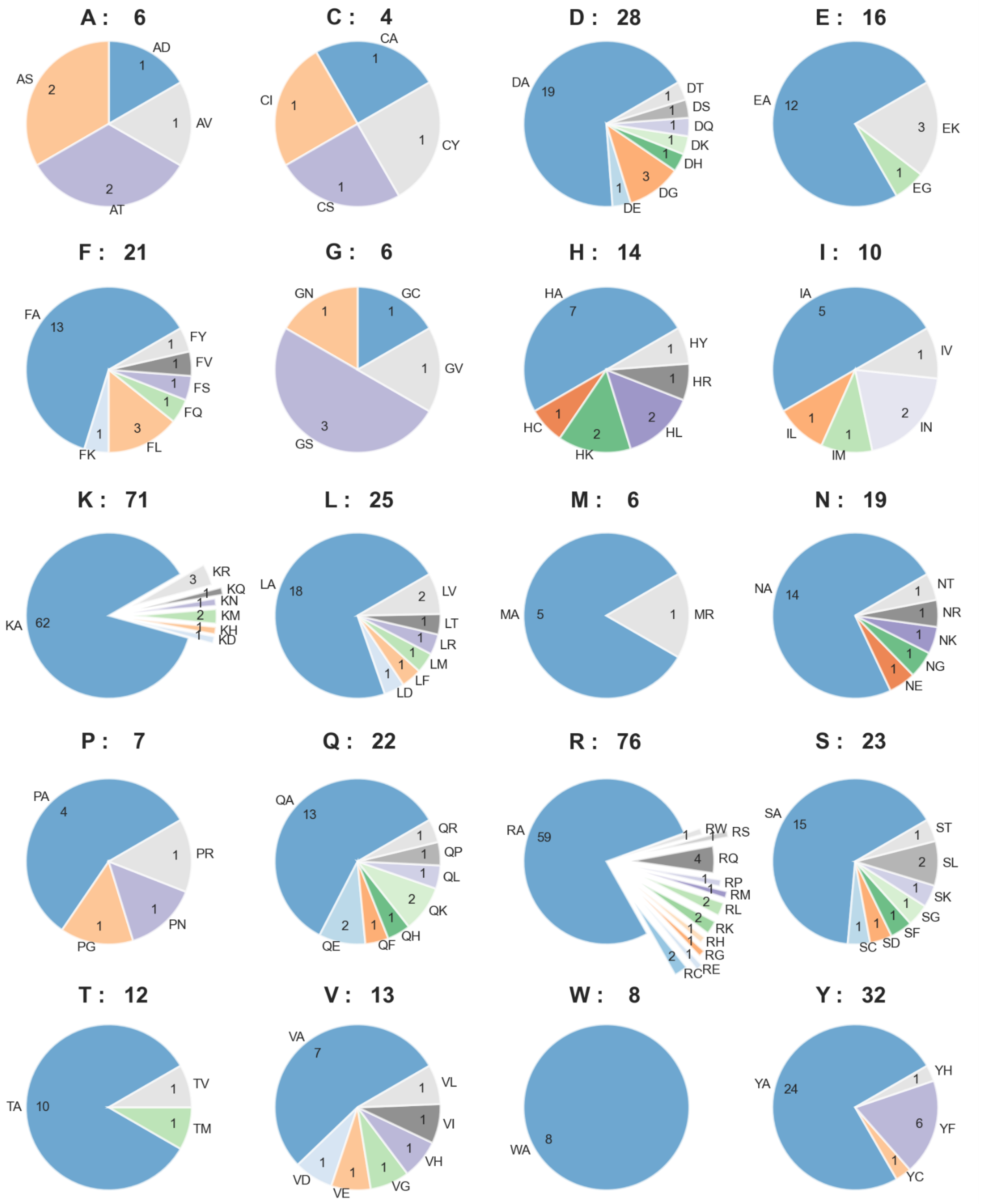

Figure S1j. Pie-chart summarizing count of different amino acid mutations in the S419 dataset. In each subplot, the title shows the wild-type amino acids followed by their count in the whole dataset, and the two-letter label of every pie denotes the one-letter codes of the wild-type and mutant amino acid pair respectively.

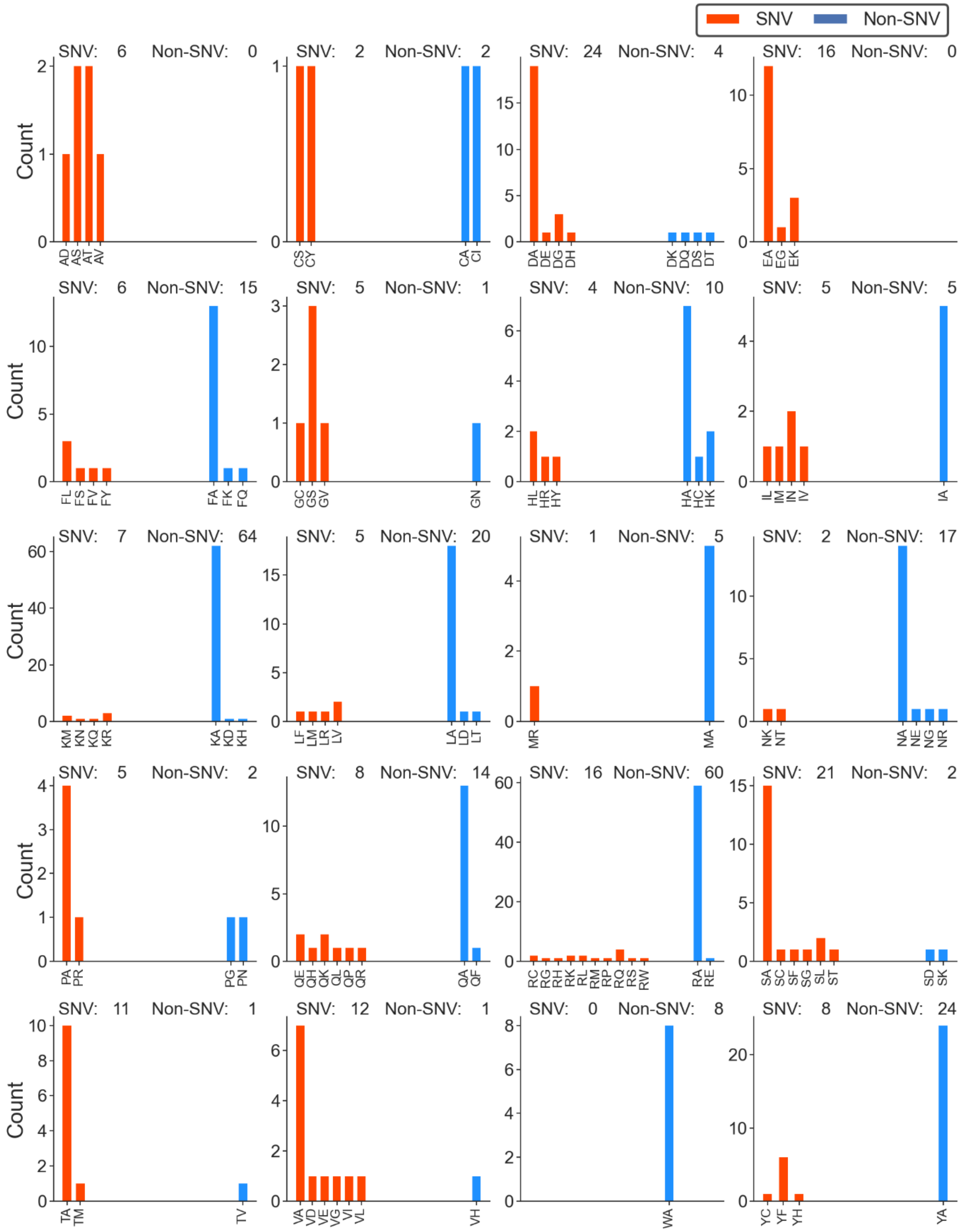

Figure S1k. Bar plots summarizing the count of SNV and non-SNV mutations in the S419 dataset. The two-letter label of every bar denotes the one-letter codes of the wild-type and mutant amino acid pairs, respectively.

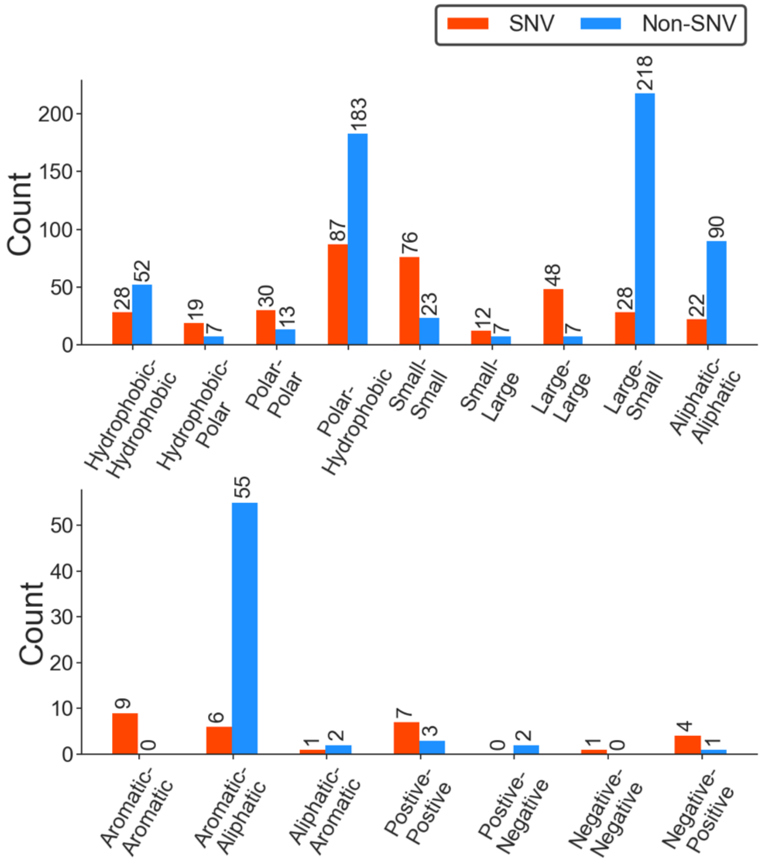

Figure S1l. SNV vs non-SNV mutations is assigned according to the type of amino acid mutation in the S419 dataset. The two-word label of bars in the plot represents the amino acid properties of wild-type and mutant residues pairs, respectively. The amino acids according to their properties are categorized as follows: Ala, Cys, Gly, Ile, Leu, Met, Phe, Pro, Trp, and Val are categorized as hydrophobic. Asp, Glu, Lys, Arg, His, Asn, Gln, Ser, Thr, and Tyr are categorized as polar. His, Phe, Trp, and Tyr as aromatic. Ala, ILe, Lys, Leu, Met, Pro, and Val as aliphatic. His, Lys, Arg as positive. Asp, and Glu as negative. Ala, Cys, Gly, Ser, Asn, Asp, Pro, Thr, and Val as small. And Arg, Gln, Glu, His, ILe, Leu, Lys, Met, Phe, Trp, and Tyr as large amino acids.

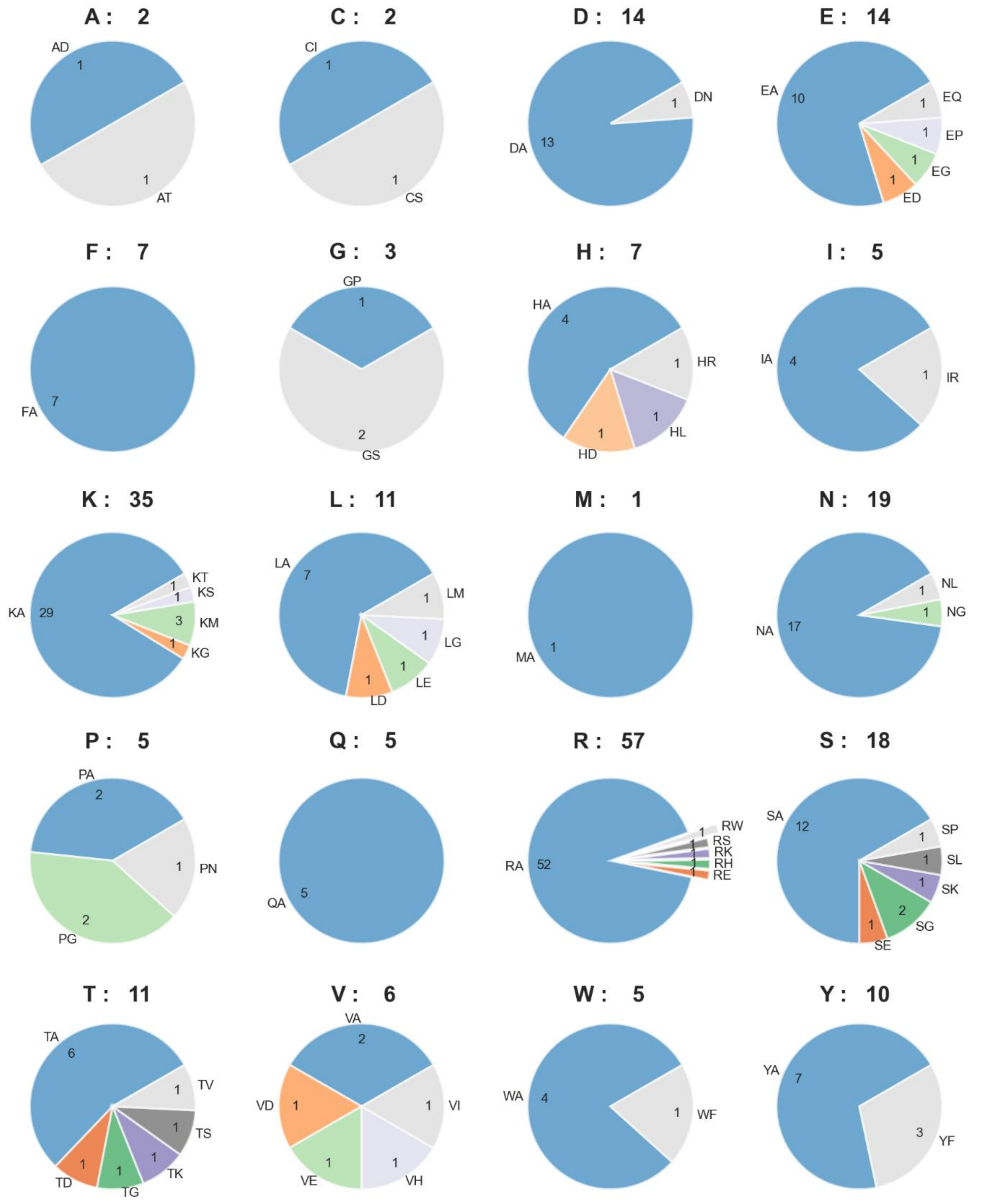

Figure S1m: Pie-chart summarizing count of different amino acid mutations in ProNAB-237. In each subplot, the title shows the wild-type amino acids followed by their count in the whole dataset, and the two-letter label of every pie denotes the one-letter codes of the wild-type and mutant amino acid pair respectively.

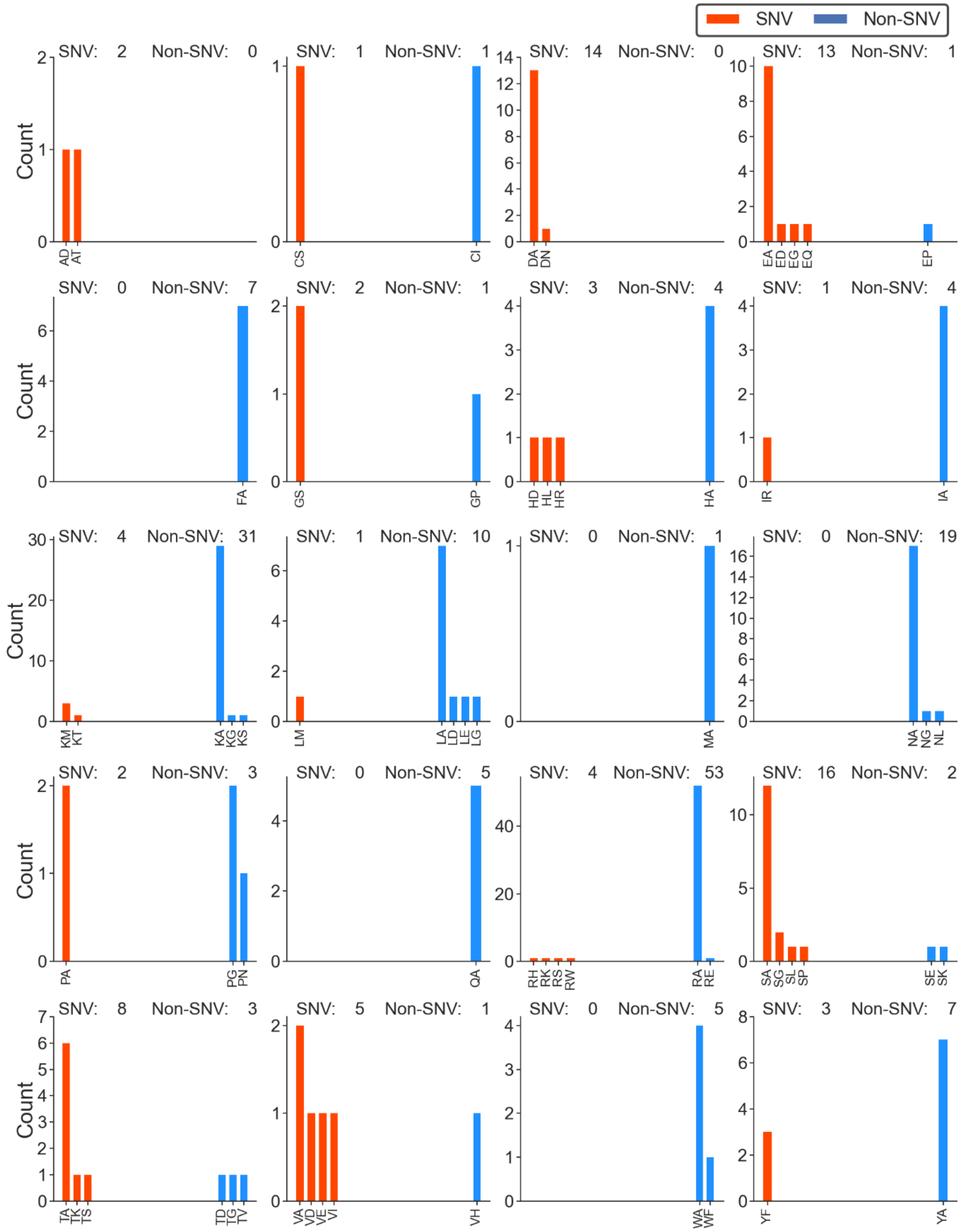

Figure S1n. Bar plots summarizing the count of SNV and non-SNV mutations in the ProNAB-237 dataset. The two-letter label of every bar denotes the one-letter codes of the wild-type and mutant amino acid pairs, respectively.

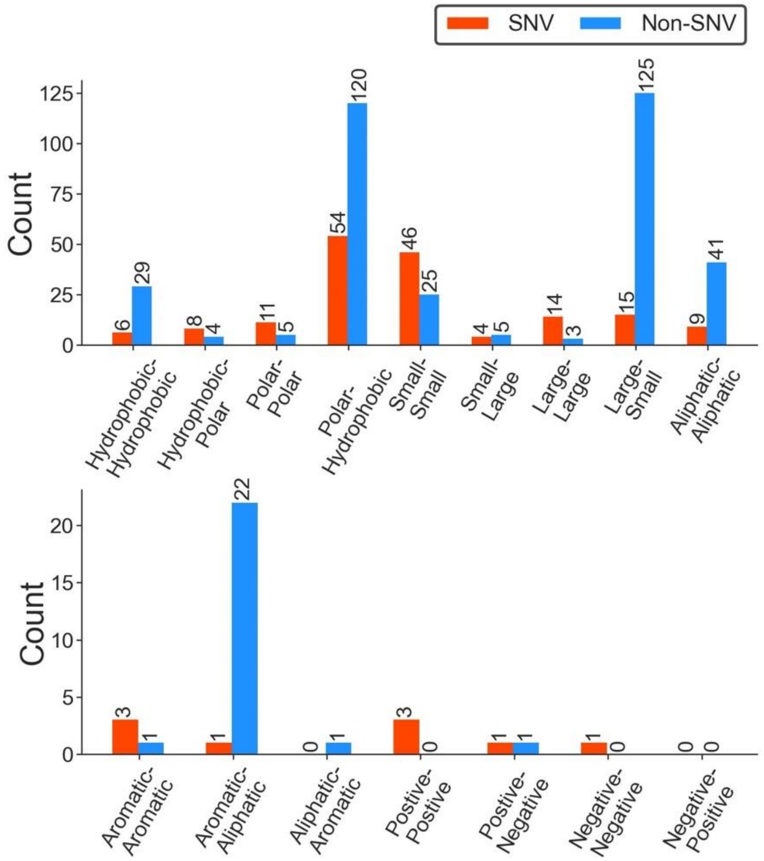

Figure S1o. SNV vs non-SNV mutations is assigned according to the type of amino acid mutation in the ProNAB-237 dataset. The two-word label of bars in the plot represents the amino acid properties of wild-type and mutant residues pairs, respectively. The amino acids according to their properties are categorized as follows: Ala, Cys, Gly, Ile, Leu, Met, Phe, Pro, Trp, and Val are categorized as hydrophobic. Asp, Glu, Lys, Arg, His, Asn, Gln, Ser, Thr, and Tyr are categorized as polar. His, Phe, Trp, and Tyr as aromatic. Ala, ILe, Lys, Leu, Met, Pro, and Val as aliphatic. His, Lys, Arg as positive. Asp, and Glu as negative. Ala, Cys, Gly, Ser, Asn, Asp, Pro, Thr, and Val as small. And Arg, Gln, Glu, His, ILe, Leu, Lys, Met, Phe, Trp, and Tyr as large amino acids.

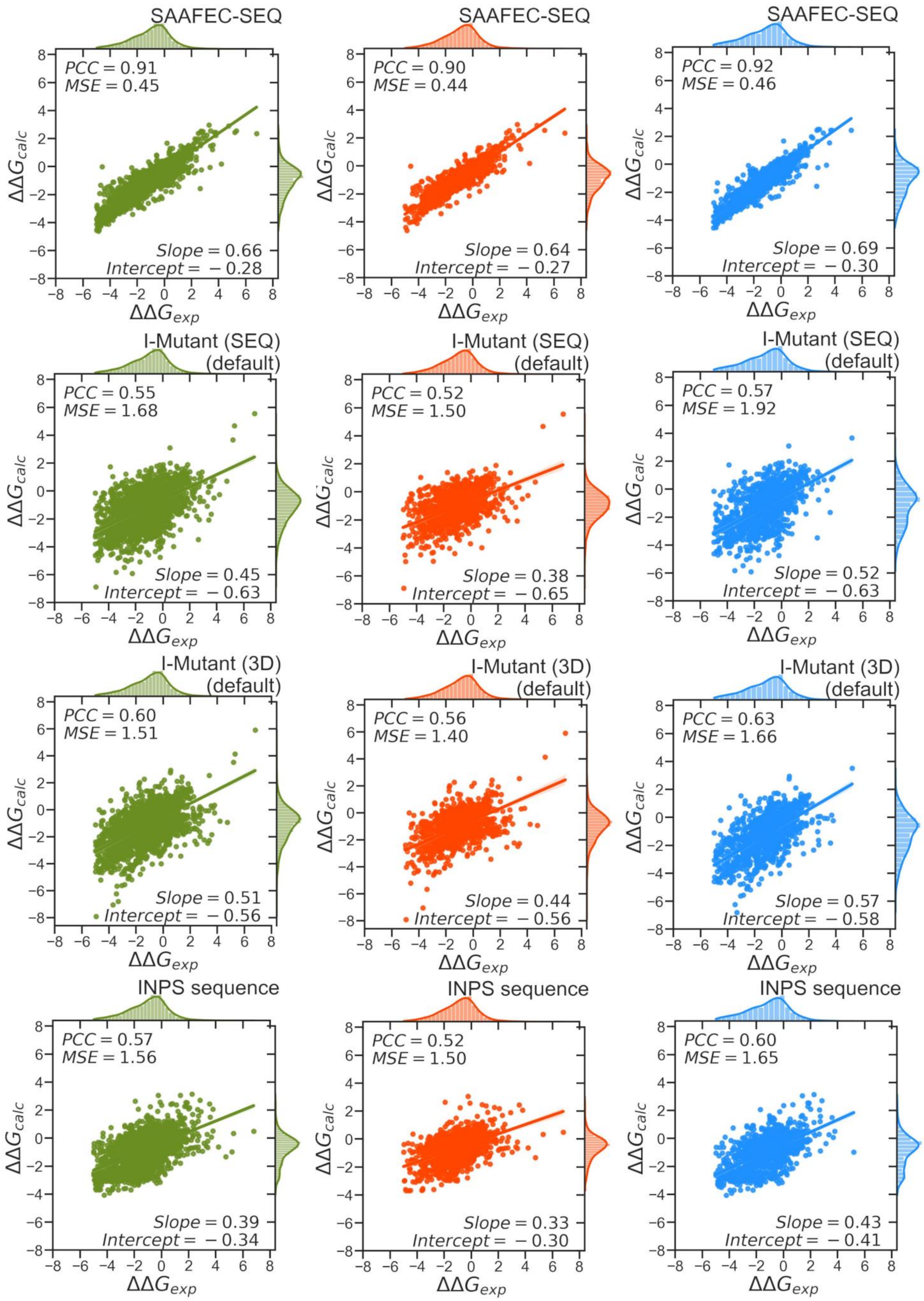

SAAFEC-SEQ
PCC = 0.91
MSE = 0.45
Slope = 0.66
Intercept = − 0.28
SAAFEC-SEQ
PCC = 0.90
MSE = 0.44
Slope = 0.64
Intercept = − 0.27
SAAFEC-SEQ
PCC = 0.92
MSE = 0.46
Slope = 0.69
Intercept = − 0.30
I-Mutant (SEQ) (default)
PCC = 0.55
MSE = 1.68
Slope = 0.45
Intercept = − 0.63
I-Mutant (SEQ) (default)
PCC = 0.52
MSE = 1.50
Slope = 0.38
Intercept = − 0.65
I-Mutant (SEQ) (default)
PCC = 0.57
MSE = 1.92
Slope = 0.52
Intercept = − 0.63
I-Mutant (3D) (default)
PCC = 0.60
MSE = 1.51
Slope = 0.51
Intercept = − 0.56
I-Mutant (3D) (default)
PCC = 0.56
MSE = 1.40
Slope = 0.44
Intercept = − 0.56
I-Mutant (3D) (default)
PCC = 0.63
MSE = 1.66
Slope = 0.57
Intercept = − 0.58
INPS sequence
PCC = 0.57
MSE = 1.56
Slope = 0.39
Intercept = − 0.34
INPS sequence
PCC = 0.52
MSE = 1.50
Slope = 0.33
Intercept = − 0.30
INPS sequence
PCC = 0.60
MSE = 1.65
Slope = 0.43
Intercept = − 0.41
ΔΔG_calc
ΔΔG_exp

Figure S2a. Plots of predicted ΔΔG against experimental ΔΔG for the S2648 dataset. The whole data is shown in green, SNV in red, and non-SNV in blue.

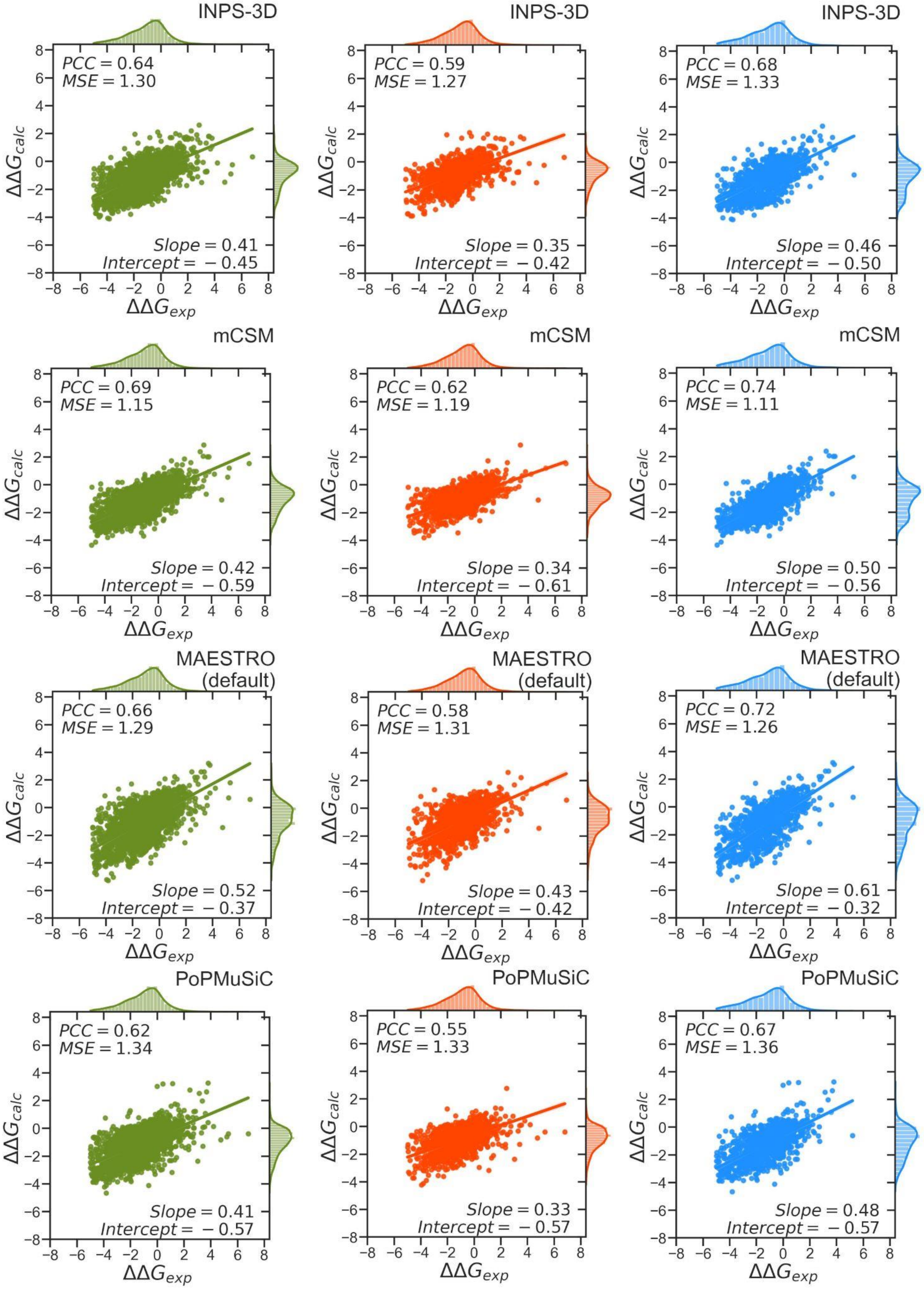

Figure S2b. Plots of predicted ΔΔG against experimental ΔΔG for the S2648 dataset. The whole data is shown in green, SNV in red, and non-SNV in blue.

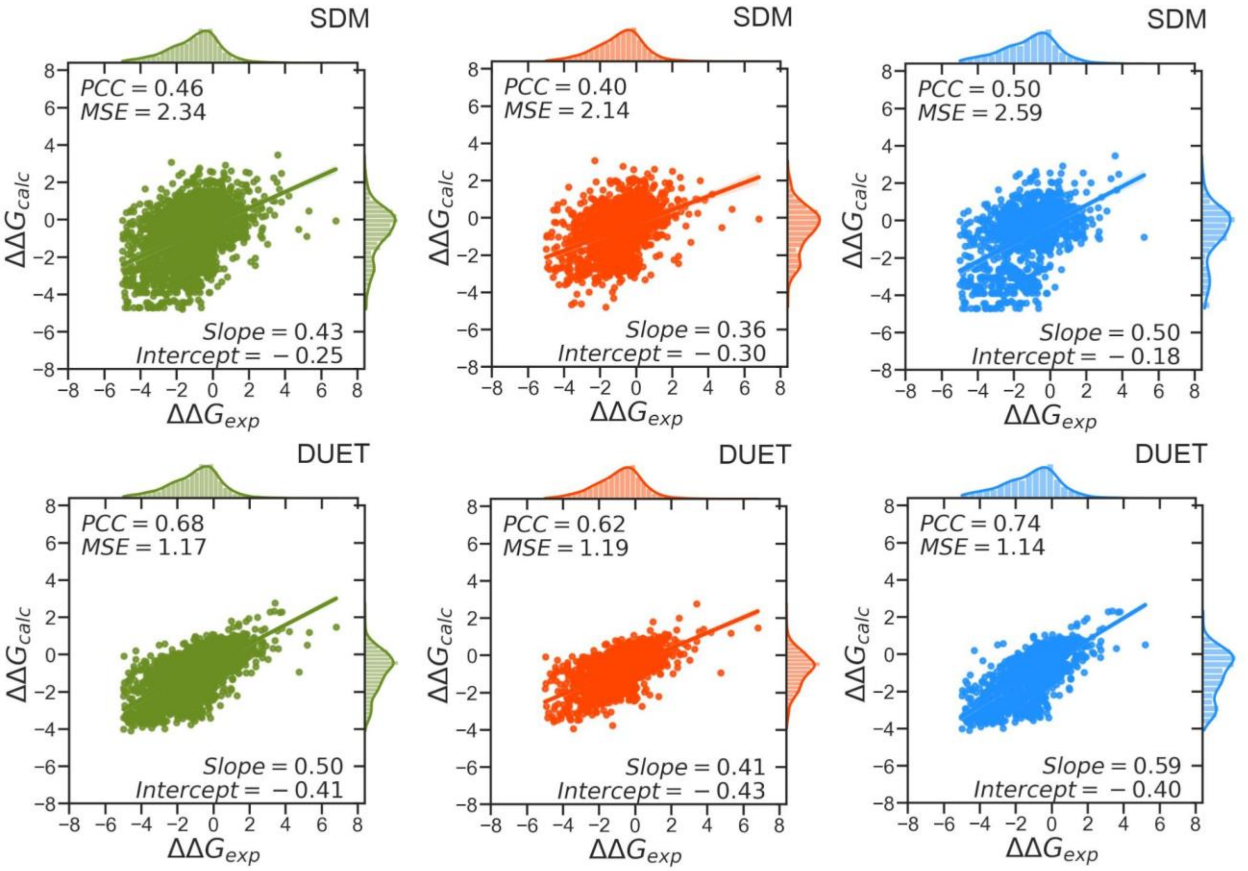

Figure S2c. Plots of predicted ΔΔG against experimental ΔΔG for the S2648 dataset. The whole data is shown in green, SNV in red, and non-SNV in blue.

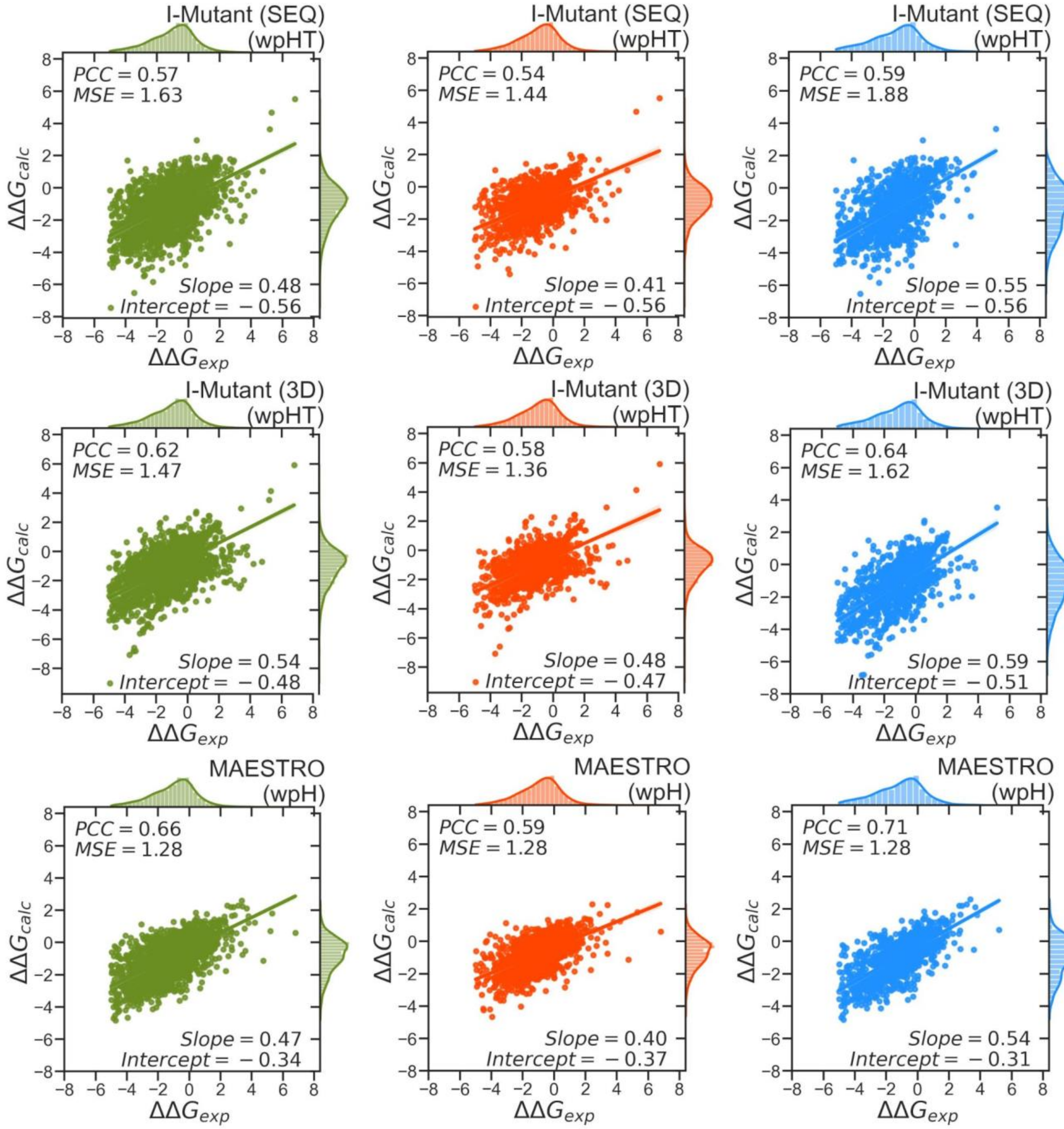

Figure S2d. Plots of predicted ΔΔG against experimental ΔΔG for the S2648 dataset. The whole data is shown in green, SNV in red, and non-SNV in blue. wpHT : the calculations have been performed using experimental pH and temperature and wpH : the calculations have been performed using experimental pH.

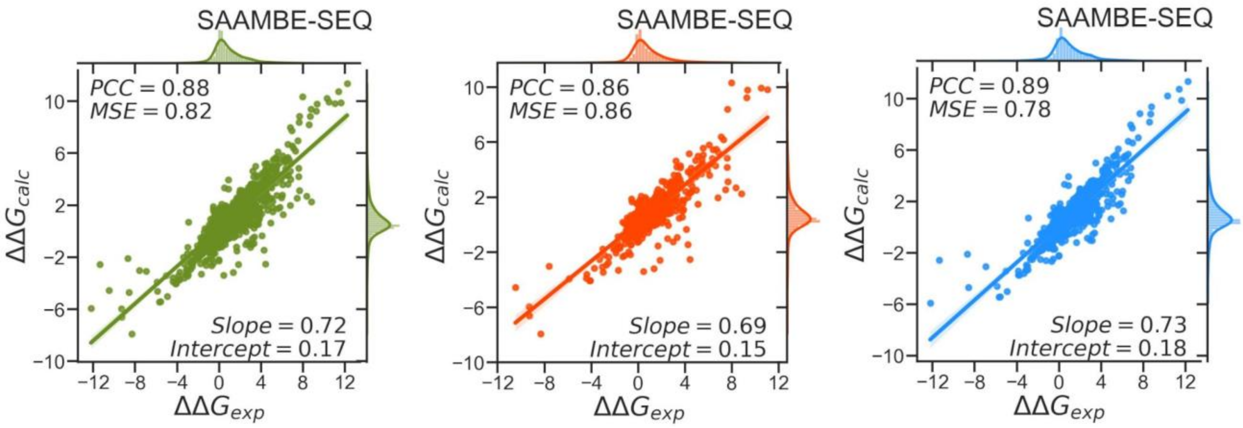

Figure S3a. Plots of predicted ΔΔG against experimental ΔΔG for the SKEMPI-SEQ-2388 dataset. The whole data is shown in green, SNV in red, and non-SNV in blue.

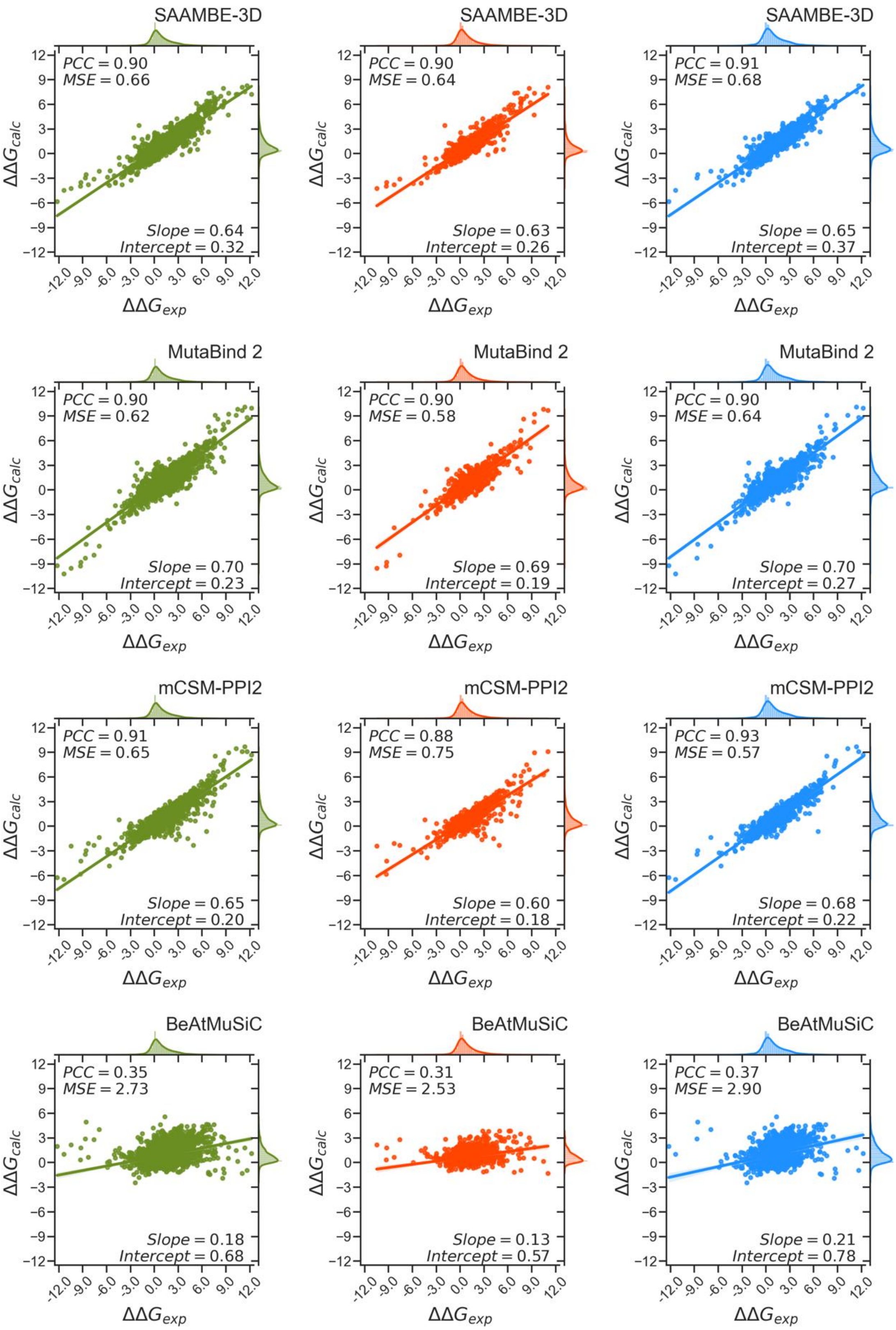

Figure S3b. Plots of predicted ΔΔG against experimental ΔΔG for the SKEMPI-3D-3775 dataset. The whole data is shown in green, SNV in red, and non-SNV in blue.

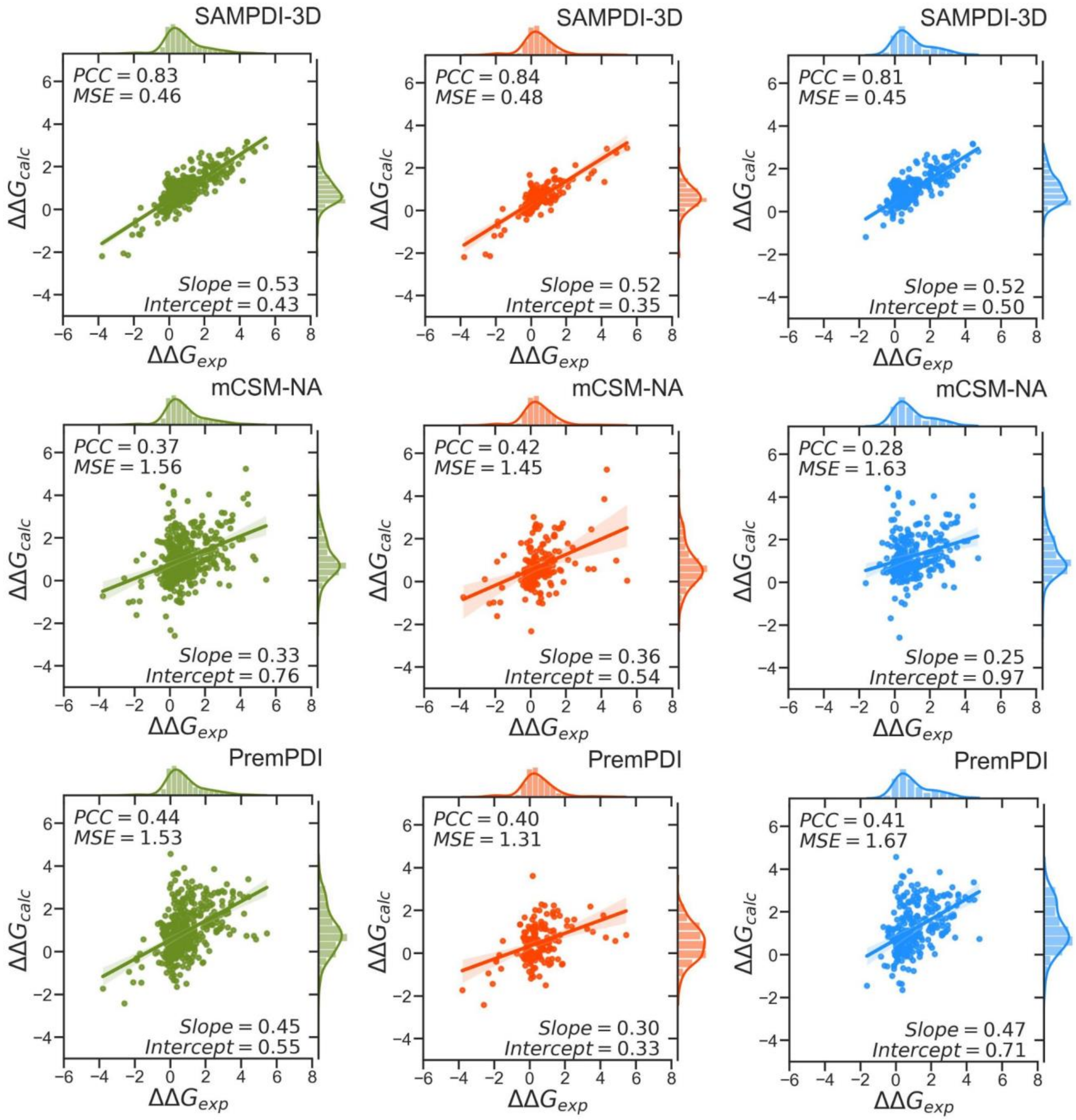

Figure S4a. Plots of predicted ΔΔG against experimental ΔΔG for the S419 dataset. The whole data is shown in green, SNV in red, and non-SNV in blue.

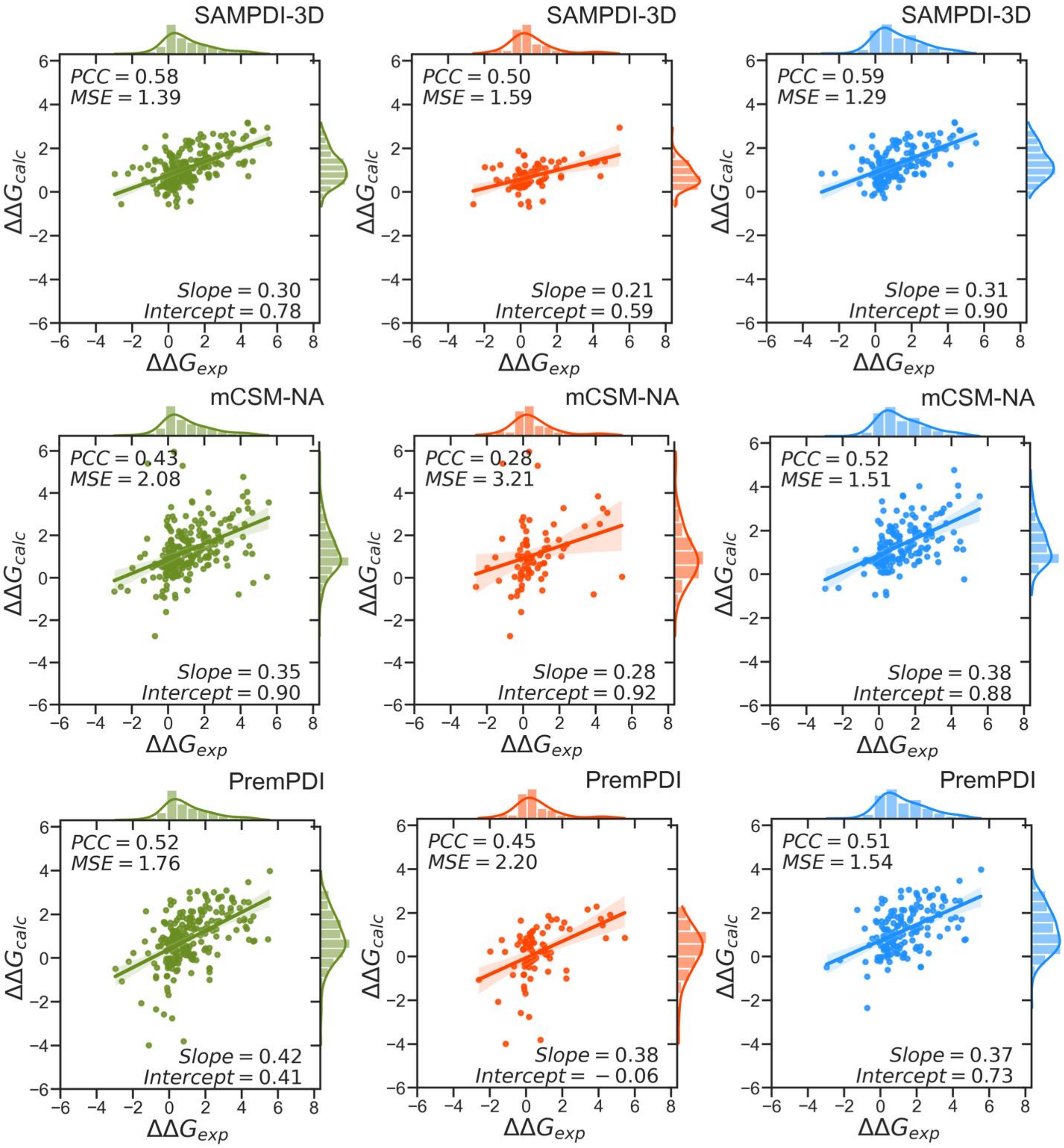

Figure S4b. Plots of predicted ΔΔG against experimental ΔΔG for the ProNAB-237 dataset. The whole data is shown in green, SNV in red, and non-SNV in blue.

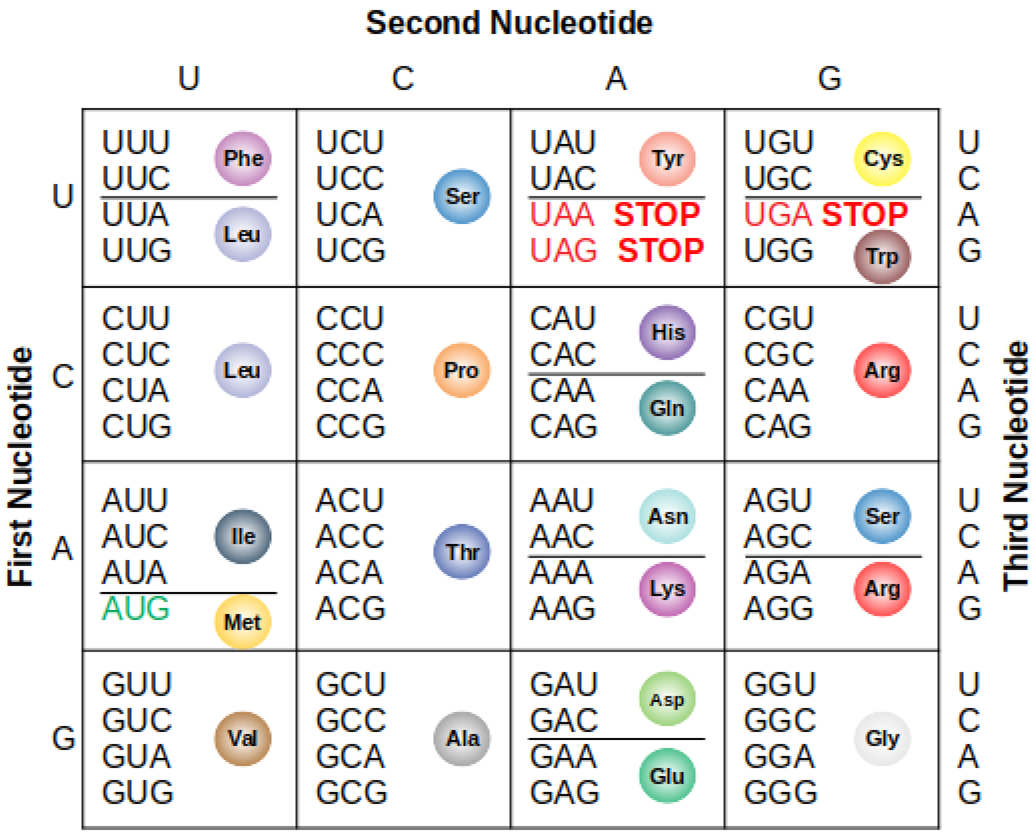

Figure S5a. Universal genetic code.

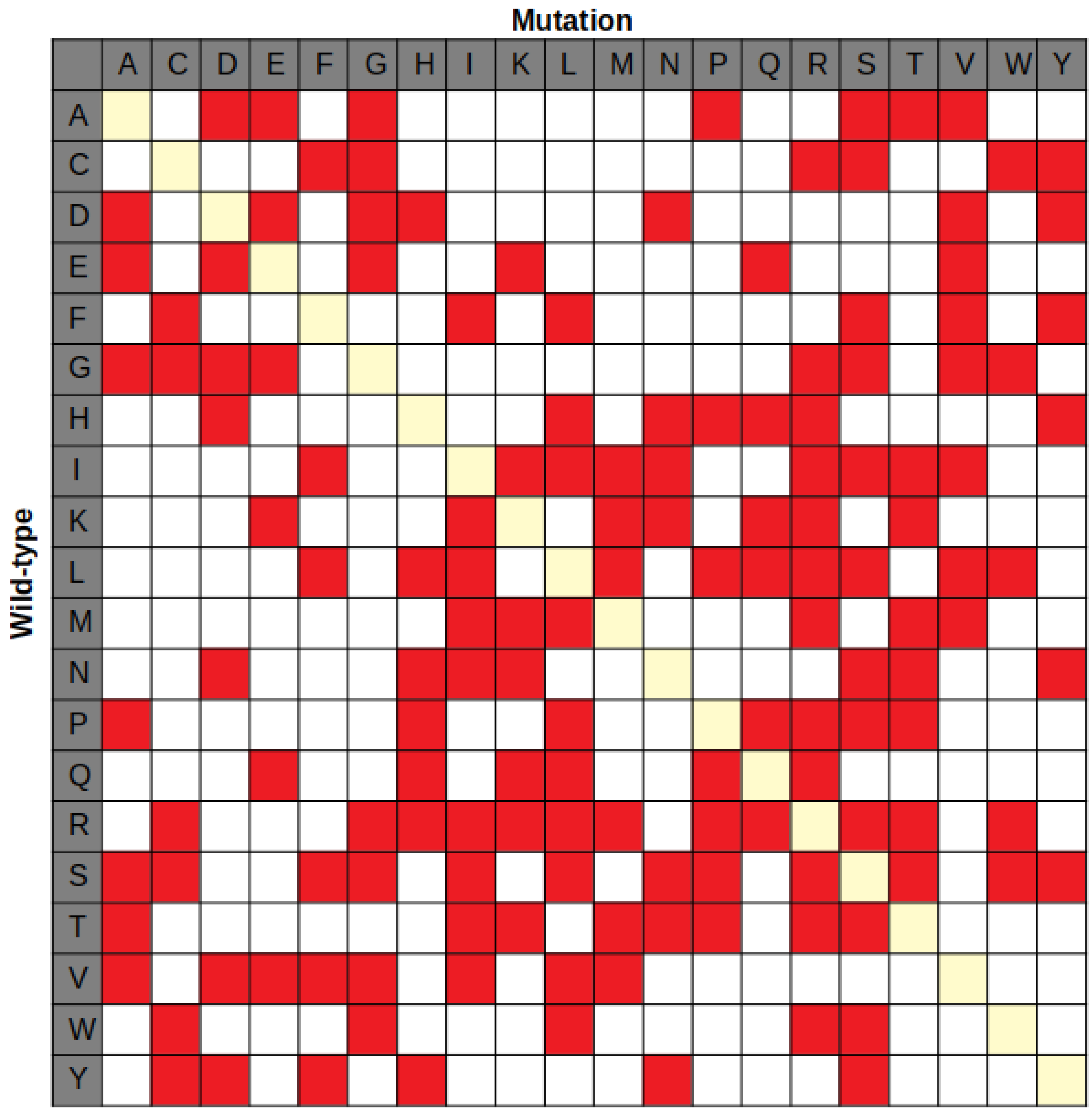

Figure S5b. Heat map showing SNV and non-SNV amino acid mutations. Red boxes indicate cases of SNVs and white cases of non-SNVs.

Supplementary Tables

Table S1. Number of SNV and non-SNV cases in the datasets.

Dataset	Total	SNV	Non-SNV
S2648	2647	1493	1154
SKEMPI-SEQ-2388	2388	1081	1307
SKEMPI-3D-3775	3775	1692	2083
S419	419	164	255
ProNAB-237	237	79	158
